# Just in Time Adaptive Interventions for Behavioral Activation in Patients with Depression (JADE): protocol of a design, feasibility and preliminary effectiveness study

**DOI:** 10.1186/s40814-026-01835-5

**Published:** 2026-05-13

**Authors:** Farid Chakhssi, Chani Nuij, Nadine Köhle, Eva Goedendorp, Jorge Piano Simoes, Alyssa Jongeneel, Gerben J. Westerhof, Jannis Kraiss

**Affiliations:** 1 Thubble/Dimence Groep, Deventer, the Netherlands; 2 Department of Psychology, Health and Technology, University of Twente, Enschede, Netherlands

## Abstract

**Background:**

Depression is a major public health problem with a rising prevalence and a negative impact on quality of life, mortality, and morbidity. While effective treatments, such as behavioral activation, are available, they are only effective for approximately half of the patients. Adherence to activities between therapy sessions is associated with better outcomes in behavioral activation, yet adherence rates could be improved. Just-in-time adaptive interventions (JITAIs) offer the potential to improve adherence to activities and thus improve outcomes in behavioral activation for depression; however, to date, they have not yet been studied.

**Methods:**

This mixed-methods study uses a participatory design phase with 12 participants with depression and 4 therapists to develop JITAIs for behavioral activation in depression, through focus groups and thematic analyses to answer the specific design question. A subsequent evaluation phase consists of a micro-randomized trial to assess the proximal effects of JITAIs and a nonrandomized quasi-experimental design to evaluate the feasibility and changes in distal outcomes compared with treatment as usual (TAU). Participants diagnosed with depression will be recruited from a mental health institution: 52 receiving TAU plus JITAIs and 52 receiving TAU only. The primary outcome is feasibility, and JITAIs are considered feasible when the dose-received adherence rate is greater than 60%. The secondary outcomes are acceptance as measured via the System Usability Scale, the Client Satisfaction Questionnaire and the Twente Engagement with E-health Technologies Scale. Ecological momentary assessments of depression and well-being are used as proximal outcomes, and the Patient Health Questionnaire-9, the Mental Health Continuum–Short Form, the Questionnaire about the Process of Recovery, and the EuroQol-5 Dimension are used as distal outcomes at the end of treatment.

**Discussion:**

This study aims to develop and evaluate JITAIs to support behavioral activation in the treatment of depression. If feasible, JITAIs have the potential to be further examined in randomized controlled trials and benefit people with depression by providing a scalable way to improve adherence and outcomes in behavioral activation.

**Trial registration:**

The Netherlands Clinical Trial Register (NL-OMON57144), prospectively registered on October 22, 2024.

**Supplementary Information:**

The online version contains supplementary material available at 10.1186/s40814-026-01835-5.

## Background

Depression is a major public health problem, with a rising prevalence and negative impact on quality of life, mortality, and morbidity [1]. Several effective treatments are available, including medication and psychological treatments. Although treatments are effective, they appear to be effective for only approximately half of the participants [2, 3]. These findings indicate that further research is needed to improve the treatment of depression.

Behavioral activation is one of the recommended psychological treatments for depression. While it is the behavioral component of cognitive behavioral therapy (CBT), it has also developed into a stand-alone treatment that can be as effective as the full CBT package [4, 5]. Behavioral activation encourages individuals with depression to seek activities that are expected to result in a sense of pleasure, mastery, or accomplishment. Examples of behavioral activities include gardening, walking, swimming, watching TV, cooking a favorite meal, reading a book, and talking about sports. Strategies such as monitoring mood, identifying pleasurable activities, and planning activities are used to achieve this goal. Meta-analyses have shown a large effect of behavioral activation on depressive symptoms [6], and several studies have shown that behavioral activation can be effectively implemented by a variety of trained disciplines [7–9].

Although behavioral activation is as effective as other treatments, including CBT, it benefits only half of the participants [2]. Participants who adhere to activity planning between sessions generally experience better treatment outcomes and fewer symptoms (small-to-moderate effect size) than those who perform fewer or no activities do [10–13]. However, adherence between sessions is far from ideal, with studies showing that only 47% of activities are completed and 27% are never attempted [14]. The main barriers to adherence were difficulty in selecting appropriate activities and problems while performing them, including perceived difficulty, lack of opportunity, and fear or avoidance [15]. From a behavioral activation perspective, such withdrawal and avoidance reduce contact with reinforcing experiences, thereby maintaining depressive symptoms. Interventions that support the start of small, feasible, and personally meaningful activities may help disrupt avoidance cycles and increase opportunities for pleasure and mastery [5]. Adherence to activities between sessions can be increased by discussing potential barriers between patients and therapists, involving patients in activity selection, and using reminders [16]. Although adherence to activities related to behavioral activation may increase its effects, it remains a significant challenge [6]. This gap in treatment adherence suggests the need for innovative approaches to support individuals more effectively between sessions in their daily lives.

Just-in-time adaptive interventions (JITAIs) offer a promising approach by providing context-specific support to improve adherence in participants’ daily lives. JITAIs use ecological momentary assessment and intervention (i.e., repeated measurements and delivery of interventions in daily life) to adapt to daily fluctuations in an individual’s state and context. The use of smartphones offers new opportunities for the real-time collection of data from everyday life, such as subjective experiences (e.g., symptoms, cognitions, emotions, and behaviors), context (e.g., location and activity), and the provision of interventions when an individual needs support [17–19]. JITAIs targeting alcohol use, physical activity, smoking, and anxiety symptoms have shown promising results [20]. However, no study has yet developed or evaluated JITAIs for behavioral activation in people with depression [21].

In our view, JITAIs are particularly suitable for behavioral activation because of the use of smartphones in everyday life, the possibility of providing personalized support with minimal or no supervision, and the use of reminders that may improve adherence. To improve our chances for the successful development of JITAIs, we will adhere to the design principles developed by Nahum-Shani and colleagues [19]. Following these principles, JITAIs for behavioral activation should consist of the following key components: decision points (e.g., moments when low mood is detected), intervention options (e.g., prompts to engage in pleasurable or meaningful activities), tailoring variables (e.g., self-reported mood tracked via momentary assessments), and decision rules (e.g., when vulnerability indicators exceed a prespecified threshold, prompt an activity suggestion). Additionally, JITAIs should target short-term goals called proximal outcomes (e.g., increased momentary activation and improved mood/well-being) that are related to long-term goals called distal outcomes (e.g., reduced depressive symptoms or increased well-being).

In this study, repeated EMA at decision points serves as the basis for tailoring: it captures momentary indicators of vulnerability and opportunity (tailoring variables). Together with prespecified decision rules, these variables determine whether an activity-suggestion prompt is delivered. These prompts are intended to facilitate the initiation of small, feasible, and personally meaningful activities, thereby interrupting withdrawal and avoidance cycles, and increasing contact with reinforcing experiences. This increased engagement is expected to incrementally improve momentary mood and well-being (proximal outcomes), and over time, lead to reductions in depressive symptoms (distal outcomes). Consistent with behavioral activation theory, the emphasis is on activation preceding mood improvement. Accordingly, activity initiation is targeted specifically during periods of vulnerability (i.e., low mood) where the risk of withdrawal or avoidance is high, rather than treating low mood as an indicator of readiness.

## Study aims and hypotheses

Because of the promising characteristics of JITAIs for behavioral activation in depression, our goal is to develop and evaluate JITAIs that target behavioral activation for depression. This will involve a design phase, in close collaboration with individuals with depression and therapists, and an evaluation phase to assess feasibility, acceptance, and preliminary effectiveness. We hypothesize that (1) it will be feasible and acceptable to deliver JITAIs to patients receiving treatment for depression, (2) JITAIs will result in improved proximal outcomes in terms of depression and well-being compared with not receiving JITAIs, and (3) behavioral activation with JITAIs will result in improved distal outcomes from baseline to post-treatment compared with TAU in terms of depression, well-being, personal recovery, and quality of life.

## Methods

### Design

This study uses a mixed-methods design that combines a participatory design phase with an evaluation phase. The design phase aims to develop JITAIs for behavioral activation in individuals with depression in collaboration with patients and therapists through focus groups. The evaluation phase consists of two components: a micro-randomized trial (MRT) to assess the proximal effects of JITAIs. In this component, randomization occurs at the within-participant level rather than between participants. Second, a nonrandomized quasi-experimental design to evaluate changes in between-group distal effects. The latter consists of a comparison of changes in mental health outcomes from baseline to post-treatment between JITAIs in addition to TAU and TAU only (comparator).

### Participants

#### Participants with depression

Participants will be recruited from a mental health institution in the Netherlands. For the design phase, a purposive sample of twelve participants with depression, including six participants with low literacy or low digital literacy, will be recruited. For the evaluation phase, 104 participants will be recruited: 52 for the treatment-as-usual with the JITAIs as add-on condition (TAU + JITAIs) and 52 for the treatment-as-usual (TAU) condition. The inclusion criteria for the participants in the evaluation phase are as follows: (a) age 18 years or older, (b) diagnosis of depressive disorder according to the DSM-5, (c) scheduled to start (cognitive) behavioral therapy for depression and (d) possession of an Android or iOS smartphone. The exclusion criterion is insufficient proficiency in Dutch to participate in the study and complete the questionnaires.

#### Therapists

The participating therapists will be recruited from the same mental health institution as the participants. Therapists include nurses, nurse practitioners, master-level psychologists, registered psychologists, or psychiatrists.

For the design phase, 4 therapists from different disciplines will be recruited. For the evaluation phase, 10 therapists will be recruited. In the evaluation phase, all participating therapists will attend a 2-h workshop explaining the JITAIs and the software application used for the JITAIs. At the end of the study, the therapists will be asked to discuss how they and their patients have experienced the JITAIs through a qualitative interview and a questionnaire [22].

## Measures

### Primary outcome

JITAIs are considered feasible when average dose-received adherence exceeds 60%, defined as the proportion of completed JITAIs among the total number of JITAIs received. This threshold was prespecified as a progression criterion for future evaluation. The percentage is based on a review of digital interventions for depression, in which a dose-received adherence higher than 60% was significantly associated with improved outcomes [23].

### Secondary outcomes

The following measures will be used to assess acceptance:

- *Client Satisfaction Questionnaire (CSQ-8)*. The CSQ-8 [24] is a questionnaire aimed at measuring satisfaction via 8 items. These items (e.g., “Would you like to use the JITAIs in the future?”) are scored on a 4-point Likert scale ranging from 1 to 4. A minimum score of 8 and a maximum score of 32 can be obtained for the CSQ-8. The scores are regarded as 'poor' when they fall between 8 and 13, ‘fair’ when they are between 14 and 19, ‘good’ when they are between 20 and 25, and ‘excellent’ when they are between 26 and 32.
- *System Usability Scale (SUS)*. The SUS [25] is a questionnaire measuring the usability of the JITAIs with 10 items. These items (e.g., are the JITAIs easy to use? and “I needed the help of a technical person to be able to use the JITAIs”) are scored on a 5-point Likert scale ranging from strongly disagree (0) to strongly agree (4). A minimum score of 0 and a maximum score of 40 can be obtained for the SUS. The total scores are multiplied by 2.5, and the total scores above 68 indicate acceptance of the system, with better acceptance scores between 70 and 90 and superior acceptance scores above 90.
- *Twente Engagement with Ehealth Technologies Scale (TWEETS)*. The TWEETS [26] is a questionnaire measuring engagement with e-health technologies with 9 items. The questionnaire consists of items on behavioral engagement (e.g., [this technology] is part of my daily routine), cognitive engagement (e.g., [this technology] makes it easier for me to work on [my goal]), and emotional engagement (e.g., I enjoy using [this technology]) and is scored on a 5-point Likert scale ranging from strongly disagree (0) to strongly agree (4). A minimum score of 0 and a maximum score of 36 can be obtained on the TWEETS, with a higher score indicating greater engagement. Participants who are engaged are more likely to have sum scores ≥ 27 [26].

JITAIs are considered acceptable when one of the following criteria is met: a CSQ-8 score ≥ 20, a SUS score ≥ 68, or a TWEETS sum score ≥ 27. The more criteria that are met, the more acceptable JITAIs are considered.

### Proximal outcomes

#### Ecological Momentary Assessment (EMA) survey

A total of 16 questions are included in the EMA questionnaire (see Table 1). The morning questionnaire begins with an assessment of rest (I feel rested) rated on a scale ranging from not at all (1) to very much (7). Four items assessing depressive symptoms, which are based on the PHQ-9, are related to mood and energy level (i.e., “I feel down”; “I feel tired”; “I feel listless”; “I feel energetic”) and two items concerning well-being (“I feel satisfied”; “I feel cheerful”) [27]. These mood and well-being items are also rated on a scale ranging from not at all (1) to very much (7). These momentary mood and energy items also serve as tailoring variables at decision points during the MRT. Importantly, these tailoring variables indicate vulnerability or opportunity states in which withdrawal or avoidance is more likely and external prompting may facilitate the start of a feasible activity, consistent with an activation-first behavioral activation model. Additionally, participants are asked about their current location (e.g., at home, at school), who they are with (e.g., alone, with family), what activity they were doing before the measurement (e.g., studying, working), and how they rate that activity on a scale from very unpleasant to very pleasant. In subsequent EMA assessments after the first one of the day, participants are asked if they performed a suggested activity (yes/no). If they answer no, they are asked for the reason (e.g., did not feel like it, did not have energy, was not convenient, or other). If they answer yes, they rate how pleasant the activity was (very unpleasant to very pleasant) and how much satisfaction it provided (none at all to very much). The evening questionnaire includes additional items: an assessment of overall activity level during the day on a scale from not active at all (1) to very active (7), an evaluation of the best moment of the day on a scale from very unpleasant to very pleasant, and an open field for comments. The evening survey serves as a reflection on the day rather than as a decision point (see procedure). Finally, daily step counts are passively collected once a day via the participants’ smartphone sensors, as higher daily step counts are an indication of improvement in depressive symptoms [28].

**Table 1:**
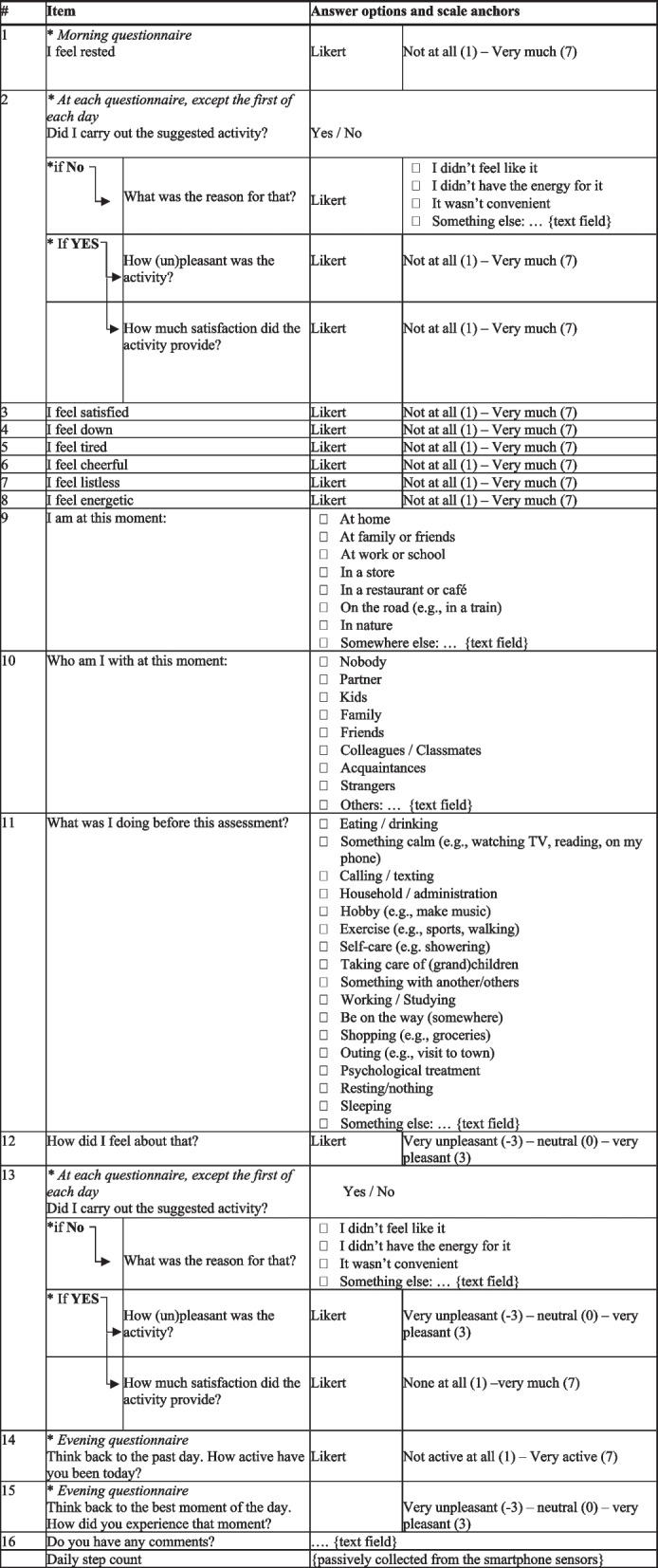
Ecological momentary assessment items

### Distal outcomes

#### Patient Health Questionnaire (PHQ-9)

The PHQ-9 [29] is a self-report questionnaire aimed at using nine items to measure the nine DSM criteria for depression. The items are scored on a 4-point Likert scale ranging from never (0) to very often (3). A minimum score of 0 and a maximum score of 27 can be obtained on the PHQ-9, with a higher score indicating more depressive symptoms. The PHQ-9 is designed and validated to detect and monitor depression [30].

#### Mental Health Continuum-Short Form (MHC-SF)

The MHC-SF [31] is a 14-item questionnaire aimed at measuring positive mental health. These items measure emotional well-being (3 items), social well-being (5 items), and psychological well-being (6 items) and are scored on a 6-point scale ranging from never (0) to every day (5). An average can be calculated on the MHC-SF, with a higher average score indicating greater well-being.

#### Questionnaire about the process of recovery (QPR)

The QPR [32] is a questionnaire aimed at measuring personal recovery with 15 items. These items (e.g., “I feel better about myself” or “I can actively engage with life”) are scored on a 5-point Likert scale ranging from disagree strongly (0) to agree strongly (4). A minimum score of 0 and a maximum score of 60 can be obtained on the QPR, with a higher score indicating greater personal recovery.

#### EuroQol-5 Dimension (EQ-5D)

The EQ-5D [33] is a self-report questionnaire designed to measure health-related quality of life across five key dimensions: mobility, self-care, usual activities, pain/discomfort, and anxiety/depression. Each of the five dimensions is rated on 5 levels of severity, with responses ranging from “no problems” to “severe problems.” The EQ-5D also includes a visual analog scale (VAS), where individuals rate their overall health on a scale from 0 (worst imaginable health) to 100 (best imaginable health).

### Procedure

The study was approved by the Medical Ethics Review Committee (NL87481.091.24), and is registered in the Dutch Trial Register (NL-OMON57144). Written informed consent will be obtained by a member of the research team before any study procedures start.

Participants will be enrolled by the research team in collaboration with the treating clinicians.

#### Design phase

The participants (patients and therapists) will attend two online focus group interviews (60 min each) to discuss the design of JITAIs for behavioral activation in depression using a topic list with prompted questions. After the focus groups, a template for the intervention message will be developed in m-Path and tested by two patients and two therapists for 2 weeks, followed by individual interviews regarding their experiences. Based on their experiences, a final version will be developed and examined during the evaluation phase. Patients will receive a remuneration of 25 euros after completing the focus group interviews.

#### Evaluation phase

The participants in the TAU + JITAI group first receive instructions on installing m-path [34] on their phones. Table 2 provides an overview of the measures and assessment times.

**Table 2:** Overview of the measures and assessment times for patients in the TAU + JITAI and TAU groups

	Baseline (week #)	Evaluation (week #)					
	1	1	2	3	4	5	6
Measures							
CSQ-8							•
SUS							•
TWEETS							•
EMA survey	•	•	•	•	•	•	•
PHQ-9	• +	• +					• +
MHC-SF	• +	• +					• +
QPR	• +	• +					• +
EQ-5D	• +	• +					• +

*CSQ-8* Client Satisfaction Questionnaire, *SUS* System Usability Scale, *TWEETS* Twente Engagement with Ehealth Technologies Scale, *EMA survey* Ecological Momentary Assessment survey, *PHQ-9* Patient Health Questionnaire-9, *MHC-SF* Mental Health Continuum-Short Form, *QPR* Questionnaire about the process of recovery, *EQ-5D* EuroQol-5 Dimension

• = TAU + JITAI assessment

+ = TAU only assessments

They will complete baseline questionnaires (PHQ-9, MHC-SF, QPR, and EQ-5D) and undergo a 1-week baseline period with two EMA surveys per day following a semi-random sampling in time blocks of 1 h (e.g., 09:00–10:00; 12:00–13:00; 15:00–16:00, and 18:00–19:00). Each assessment takes place at random moments in one of the time blocks, and scheduling of the time blocks is decided by the participants at the start of the baseline. After the baseline period, the intervention phase (MRT) will start and last 6 weeks. During the MRT, participants receive four EMA surveys per day—following semi-random sampling in time blocks of 1 h—which serve as a decision point. At each decision point, the participant is eligible for randomization if they (1) completed the EMA and (2) met a prespecified tailoring-variable threshold indicating that they are in the symptomatic half of the item scale (i.e., a vulnerability state for withdrawal/avoidance). Because the EMA includes both negatively and positively valenced items, thresholds are operationalized in a directionally consistent way: for negatively valenced symptom items (e.g., “I feel down,” “I feel tired,” “I feel listless”), eligibility is defined as a score ≥ 4; for positively valenced functioning items (e.g., “I feel energetic”), eligibility is defined as a score ≤ 4. This threshold was selected to identify "moderate-or-worse" vulnerability states where the risk of avoidance is elevated [35] while managing participant burden by preventing prompting during mild or asymptomatic states. If the decision point is eligible for randomization, there is a 50% probability of receiving a JITAI and a 50% probability of receiving no JITAI. Thus, randomization occurs repeatedly within participants across eligible decision points. Allocation to the TAU + JITAI and TAU groups is not randomized. Within the TAU + JITAI condition, randomization at eligible decision points is generated automatically by m-path (simple randomization), and recruiters do not have access to future assignments. Because of the nature of the intervention, participants, therapists, and researchers are not blinded to study condition; therefore, no formal blinding or unblinding procedures apply.

For each participant, the tailoring variables will represent (1) one of the EMA items assessing depressive symptoms that exhibited the highest average score during their baseline period, and (2) one of the EMA items assessing depressive symptoms that the participant rated as “most impactful” on their daily functioning identified via a shared decision-making procedure following the baseline week. Specifically, a researcher and the participant jointly review visualized baseline EMA data (e.g., timeline plots of the symptom items), and the participant identifies which symptom most hindered their daily functioning and/or engagement in planned activities. This self-identified symptom is operationalized as the “impactful” tailoring variable. At the end of each study day, participants will receive an EMA survey that does not serve as a decision point. Two questions are added to the evening survey (activity level and best moment of the day). Importantly, the JITAIs are embedded in a therapist-supported treatment. During weekly therapy sessions, the EMA data collected during the week are visualized (e.g., timeline plots of mood and activities) and reviewed collaboratively by the therapist and participant. This integration aims to support engagement. The questionnaires completed at baseline (PHQ-9, MHC-SF, QPR, and EQ-5D) will be administered again at the start of the JITAI phase and at the end of treatment. In addition, at the end of treatment, participants will complete questionnaires regarding the acceptance of the JITAIs (CSQ-8, SUS, TWEETS). Participants in the TAU group will complete these questionnaires (PHQ-9, MHC-SF, QPR, EQ-5D) at the same time points. Participating patients in the TAU + JITAI group will receive a remuneration of 25 euros when they have completed the questionnaires at the last time point. Therapists will be interviewed at the end of treatment to share their experiences with patients using JITAIs.

### Activities

Based on the EMA survey four times a day, a decision will be made to randomize a participant to a JITAI or no JITAI based on the decision rules above. If participants are randomized to a JITAI, a message with a suggestion for an activity will be sent using m-path. The activities that will be suggested come from a “list of pleasurable activities”, which are part of the protocolized cognitive behavioral treatment for patients with a depressive disorder [36]. Examples of these activities include reading, singing, listening to music, walking, and gardening. These activities are divided into the following categories of life areas used in behavioral activation treatment protocols [5]: relationships, education/career, recreation/interests, mind/body/spirituality, and daily responsibilities. During the baseline period, participants rate how pleasurable or satisfying each activity on the list is on a scale ranging from not (0) to somewhat (1) to yes (2). Activities that are rated ≥ 1 on pleasure or satisfaction by the participant will have a follow-up question regarding the location (can this activity be performed when you are at home, outside or both?). For each participant, activities that they rated as pleasurable or satisfying and that can be performed at home, outside, or both will be selected as their personalized activities. Participants will also be prompted to choose at least one activity from each life area category. If randomized to receive a JITAI, participants will be asked which life category they prefer. After they indicate their preference, an activity will be randomly selected from their personalized activities in that category. The template used in m-path to suggest the activities will be evaluated during the course of the study and adapted based on feedback from the participants.

### Data management

Data from questionnaires and EMA assessments will be collected digitally and stored in a coded dataset accessible only to the research team. Interview transcripts will be pseudonymized. The key linking codes to identifying information will be stored separately. Data will be checked for completeness and consistency before analysis.

## Data analyses

### Design phase

#### Focus groups

The focus group data collected in the design phase will be thematically analyzed by two raters. The raters will first familiarize themselves with the data by carefully reading the focus group transcripts. They then will generate initial codes by identifying key concepts and ideas expressed by participants. Next, the raters will work together to group similar codes into potential themes. The raters will discuss discrepancies in their coding and theme development to reach consensus. Key themes that emerge from the analysis will be presented and serve as a basis for the m-path template. The experiences of the two patients and two therapists during the test will also be summarized, presented, and used to further improve the m-path template.

### Evaluation phase

#### Feasibility

Feasibility will be analyzed via descriptive statistics. We will calculate the dose-received adherence (= number of completed JITAIs divided by the total number of received JITAIs) for the complete length of the MRT, as the primary outcome. Additionally, we will divide the length of the MRT into four intervals of equal duration and calculate the dose-received adherence for each interval. This would provide more details regarding dose-received adherence during the study period.

#### Acceptance

Acceptance will be analyzed via descriptive statistics for the questionnaire data (CSQ, SUS, and TWEETS) and thematic analysis of the interview data.

#### Proximal outcomes

The effects of the JITAIs on the proximal outcomes will be analyzed with mixed-effects models. This type of model considers the nested structure of the data (e.g., multiple observations per individual per day) and adequately handles missing data [37]. In our primary analysis, we will examine the average proximal effect of delivering a JITAI (i.e., a message with an activity prompt) versus not delivering a JITAI (i.e., no message) on the depression scores (i.e., tailoring variables; average of the depression items), well-being scores (i.e., average of the two wellbeing items) at the next EMA and the daily step count, across participants and across randomized decision points. In a secondary analysis, we will examine whether the proximal effects of the JITAIs vary with time by examining the linear time trend of delivering a JITAI, assessed by estimating the interaction between the proximal effects of the JITAIs and the study day. Furthermore, moderation analyses will be conducted to evaluate whether the effect of JITAIs on the proximal outcomes depends on contextual factors by calculating the interaction effects between the intervention and contextual factors (i.e., situation: alone, with other people, at home, or outside). Sensitivity analyses will be performed by adding patient sex, age, symptom severity at baseline, and EMA survey depression item scores as covariates to the unadjusted model.

#### Distal outcomes

For the distal outcomes (PHQ-9, MHC-SF, QPR, and EQ-5D), propensity score matching will be used to match participants in the TAU group with those in the TAU + JITAI group. Propensity scores are calculated via logistic regression analysis [38], which models the probability of receiving treatment (TAU + JITAIs versus TAU) as a function of baseline covariates, including sex, age, and severity of depressive symptoms at the start of treatment. After the propensity scores are calculated, participants from the TAU group are matched with participants from the TAU + JITAIs group via a matching method (nearest neighbor matching with a caliper). This method ensures that each participant in the TAU + JITAIs group has a corresponding participant in the TAU group with a similar propensity score, making the groups similar in terms of the matched covariates. The matched sample is then used in a mixed-effects model that considers the paired nature of the matched sample by including the matched pairs as random effects. This ensures the correct estimation of standard errors and appropriate interpretation of the results. The fixed effects in the model include time (baseline, 1-week and 6-week assessment), group status (TAU + JITAIs versus TAU), and their interaction, whereas the random effects model includes variability between matched pairs and within individual participants over time. A significant interaction suggests that changes in the outcomes (PHQ-9, MHC-SF, QPR, and EQ-5D) differ between groups.

Participants may pause or discontinue the JITAI component at their own request, at the advice of the treating clinician. Data collected until then will be used for analyses, and participants will be asked to complete the outcome questionnaires. This will not affect access to TAU, and no protocol-driven modification of TAU is planned. Possible harms are expected to be limited and may include burden, annoyance, or temporary distress related to EMA or prompts. Any adverse events will be documented and discussed with the treating clinician.

#### Sample size

The primary feasibility outcome is dose-received adherence, defined as the proportion of completed JITAIs among all received JITAIs during the MRT phase. A sample size calculation for this outcome was based on the precision of the estimated proportion of completed JITAIs among received JITAIs [39]. Because adherence was defined at the JITAI level, with repeated observations nested within participants, the calculation was adjusted for within-participant correlation using a design effect [40]. During the 6-week MRT phase, participants can have up to 168 decision points (42 days × 4 per day). Assuming participants are available at 66% of these moments and a probability of 25% of receiving a JITAI at a given decision point, participants are expected to receive approximately 28 JITAIs on average. Assuming an adherence proportion of 0.60, a two-sided 95% confidence interval, an average of 28 received JITAIs per participant, and a within-participant intraclass correlation of 0.05, 46 participants would yield an approximate confidence interval half-width of 0.041.

We set the recruitment target for the TAU + JITAI group at 52 participants, comprising the 46 participants indicated by the feasibility-based precision estimate plus 6 additional participants to allow for 13% dropout observed in samples with depression [41]. For consistency in the nonrandomized comparison, we will recruit an equal number of participants in the TAU group. The proximal and distal outcome analyses are intended to provide preliminary estimates to inform a future trial.

### Protocol amendments

Important protocol modifications will be submitted to the medical-ethics committee where required, updated in the trial registry as applicable, and reported in future publications.

## Discussion

The current study will design JITAIs for behavioral activation in patients with depression and assess their feasibility, acceptability, and preliminary effectiveness. This study aims to develop and examine an innovative approach that may improve adherence to activities related to behavioral activation. If the results of the study suggest that JITAIs are regarded as feasible with > 60% dose-received adherence and acceptable, the intention is to examine JITAIs in a larger trial in the future.

Although it is not recommended to examine effectiveness in feasibility studies [42], we also aim to examine the preliminary effectiveness of JITAIs on proximal outcomes in the current study. For innovative approaches, such as JITAIs, any preliminary estimates of proximal effectiveness are informative for larger trials with more rigorous designs. However, the sample size may not be sufficient for the nonrandomized quasi-experimental design to evaluate changes between groups and increases the likelihood of Type II errors in the between-group comparisons.

In conclusion, there is a need to improve adherence to activities related to behavioral activation in individuals with depression, which may increase its effectiveness. JITAIs offer the potential to improve adherence to activities and have not yet been examined. This study is the first to develop and assess JITAIs for behavioral activation, and will provide data for further research with more rigorous research designs.

### Dissemination

The results of this study will be disseminated through peer-reviewed publication, scientific presentations, and, where feasible, a summary for participating clinicians and patients. Trial information and study status will also be updated in the trial registry as applicable.

## Supplementary Information

Supplementary Material 1.

## Data Availability

All relevant data are within the paper and its supporting information files.
